# Molecular evidence of the amelioration of toluene induced encephalopathy by human breast milk mesenchymal stem cells

**DOI:** 10.1038/s41598-022-13173-6

**Published:** 2022-06-02

**Authors:** Omaima I. Abdel Hamid, Ayat M. Domouky, Yara M. El-fakharany

**Affiliations:** 1 Forensic Medicine and Clinical Toxicology Department, Faculty of Medicine, Zagazig University, Alsharquiah, 44519 Egypt; 2 Human Anatomy and Embryology Department, Faculty of Medicine, Zagazig University, Alsharquiah, 44519 Egypt

**Keywords:** Biological techniques, Cell biology, Neuroscience, Stem cells, Neurology

## Abstract

Toluene was widely used volatile organic compound that accumulates in tissues with high lipid content. Stem cells have been proposed as an increasingly attractive approach for repair of damaged nervous system, we aimed to evaluate the ability of breast milk mesenchymal stem cells (MSc) to ameliorate toluene-induced encephalopathy. Sixty adult male albino rats were assigned to 3 groups, control, toluene, and toluene/breast milk-MSc. Neurological assessment was evaluated as well as serum levels of glial fibrillary acidic protein (GFAP), tumor necrosis factor-alpha (TNF-α), nerve growth factor (NGF), vascular endothelial growth factor (VEGF), tissue dopamine and oxidative markers. Gene expression of peroxisome Proliferator-Activated Receptor-Gamma (PPAR-ɣ), nuclear factor-kappaB (NF-kB), and interleukin-6 (IL-6) were evaluated. Moreover, histological and immunohistochemical investigation were done. Results revealed that toluene caused cerebral injury, as evidenced by a significant increase in serum GFAP, TNF-α, malondialdehyde (MDA) and nitric oxide (NO), a significant decrease in serum NGF, tissue dopamine and oxidative markers, besides, a non-significant change in VEGF. Toluene also caused changes in normal cerebral structure and cellular degeneration, including a significant decrease in the total number of neurons and thickness of frontal cortex. Meninges showing signs of inflammation with inflammatory cell infiltration and exudation, a significant decrease in MBP immunoreactivity, and increase in the percent of high motility group box protein-1 (HMGB1) positive cells. PPAR- ɣ, NF-kB, and IL-6 gene expression were all considerably elevated by toluene. These changes were greatly improved by breast milk MSc. Therefore, we conclude that breast milk MSc can attenuate toluene-induced encephalopathy.

## Introduction

Toluene (C6H5CH3), also known as methyl benzene, phenyl methane, and toluol, was a well-known clear, colorless aromatic hydrocarbon having a sweet and pungent odor^1^. Toluene was a volatile organic compound that was widely used and produced in huge amounts for use in a broad range of industrial and commercial applications including paints, inks, adhesives, varnishes, plastics, thinners, leather, and dyes^2^.

Toluene has a very high lipophilic activity. It was absorbed by inhalation, ingestion, and, to a lesser extent, cutaneous absorption. It was promptly supplied to highly perfused organs including the brain and liver, where it accumulates in lipid-rich tissues^1^. The liver was the key organ responsible for toluene metabolism, whereas the kidney was the primary organ responsible for excretion^3^.

Toluene-containing solvent misuse or occupational contact both result in high concentration toluene exposure, however solvent abusers were often exposed to greater levels than occupational exposures^4^. Furthermore, the usage of common home items (nail polish, paints, paint thinners, adhesives, synthetic odors) and cigarette smoking create the highest amounts of toluene in indoor air^5^. Toluene poisoning was frequent following environmental, unintentional, or purposeful exposures^6^.

Toluene was toxic to several organs, including the liver, kidneys, lungs, and heart^7^. However, in both humans and animals, the central nervous system (CNS) was the principal target for both acute and chronic toluene toxicity^8^. Chronic exposure to toluene damages the CNS, resulting in toluene-induced leukoencephalopathy, a common medical condition around the world^9^. Other clinical signs, such as Parkinson's disease, ataxia, psychiatric illnesses, tremors, and temporal lobe epilepsy, may differ based on the damaged brain regions^10^.

Unfortunately, toluene-induced neuronal damage seems to be permanent, and now there was no treatment available other than abstinence or nonspecific neurotrophic medicines^9^. In the adult brain, a tiny number of stem cells were located in definite areas, but this genuine stem cell pool was modest and does not play a significant role in tissue repair^11^. Stem cell transplantation has recently been recognized as an increasingly appealing method of developing therapeutics for injured nervous system repair^12^. In animal models of acute brain injury, strong data has accumulated in recent years revealing the capacity of diverse stem cell types to cause regeneration for example following acute traumatic brain injury or neurovascular insults^13^, primary degenerative CNS illnesses as Parkinson’s disease, Alzheimer’s disease, Huntington’s disease^14^. Mesenchymal stem cell therapy exhibited neuroprotective effects against toxin induced hypoxic brain injury as that caused by sodium nitrite^15^.

Up to our knowledge, no previous studies were performed to test the role of mesenchymal cell therapy in treating toluene-induced encephalopathy. In the current work, the hypothesis of the ability mesenchymal stem cells to treat toluene-induced encephalopathy will be tested.

## Materials and methods

### Chemicals

Toluene [C6H5CH3]: anhydrous colorless liquid substance with a pungent aromatic odor, molecular weight 92.13 g/mol, purity of 95%, CAS No. was 108-88-3, was purchased from El-Gomhouria Company for pharmaceutical, Egypt. 1 gm. toluene fluid was dissolved in 100 gm. olive oil to prepare 1% Tol solution, 1% Tol was the commonest dose to which industrial workers exposed to^16^. Dulbecco’s Modified Eagle Medium (DMEM): 4.5 g/L glucose, with L-glutamine (Cat no. 12-604Q) was purchased from Lonza Bioproducts Walkersville, MD 21,793–0127 USA. Penicillin–Streptomycin-Amphotericin B mixture: 10,000 units/ml Potassium Penicillin, 10,000 µg/mL Streptomycin Sulfate and 25 µg/ml Amphotericin B cat no. 17-745E used as 10 ml/L from Lonza Bioproducts Walkersville, MD 21793-0127 USA. Trypsin/EDTA solution: 0.25% trypsin containing 0.02% EDTA cat no. CC-5012from Lonza Bioproducts Walkersville, MD 21793-0127, USA.

### Human breast milk mesenchymal stem cells collection

#### Ethical approval

Milk samples were collected from the Pediatrics Department, Zagazig University Hospitals in accordance with relevant guidelines and regulations. All experimental procedures were approved and formed in accordance with the guidelines of Institutional Review Board (IRB) of Faculty of Medicine, Zagazig University, Egypt. An informed written consent was collected from twenty healthy nursing mothers. Ten samples were collected from each mother, two to five days post-partum under aseptic medical conditions.

### Human breast milk mesenchymal stem cells preparation and culture (MSc)

Following the method explained by Patki, Kadam^17^, human breast milk from each mother was centrifuged individually at 285×g for 10 min after dilution 1:2 with DMEM solution containing the penicillin–streptomycin-Amphotericin mixture. Washing the cell pellet with sterilized phosphate-buffered saline (PBS) was performed twice then DMEM media with fetal bovine serum (FBS 10%) (Biochrom AG, Berlin, Germany) supplemented with penicillin–streptomycin-Amphotericin B mixture was used to seed the cells in a 25-cm^2^ tissue culture treatment dish. The flasks were then incubated in a CO_2_ incubator (Heraeus, Hanau, Germany) at 95% relative humidity and 37 °C under 5% CO_2_. As the first passage, the medium was substituted every 2 days for 14 days. Trypsin/EDTA solution was used to trypsinize the cells at 37 °C and 80% confluency for 5 min then centrifugation at 2400×g for 20 min was done, then the cells were passaged at the 3rd passage for use in the research by harvesting with trypsin/EDTA solution and washing twice with PBS then counted by hemocytometer. Trypan blue exclusion was used to determine cell viability, with live cells being unstained while dead cells appear dark blue.

### Identification of breast milk mesenchymal stem cells (MSc)

**Figure 1:**
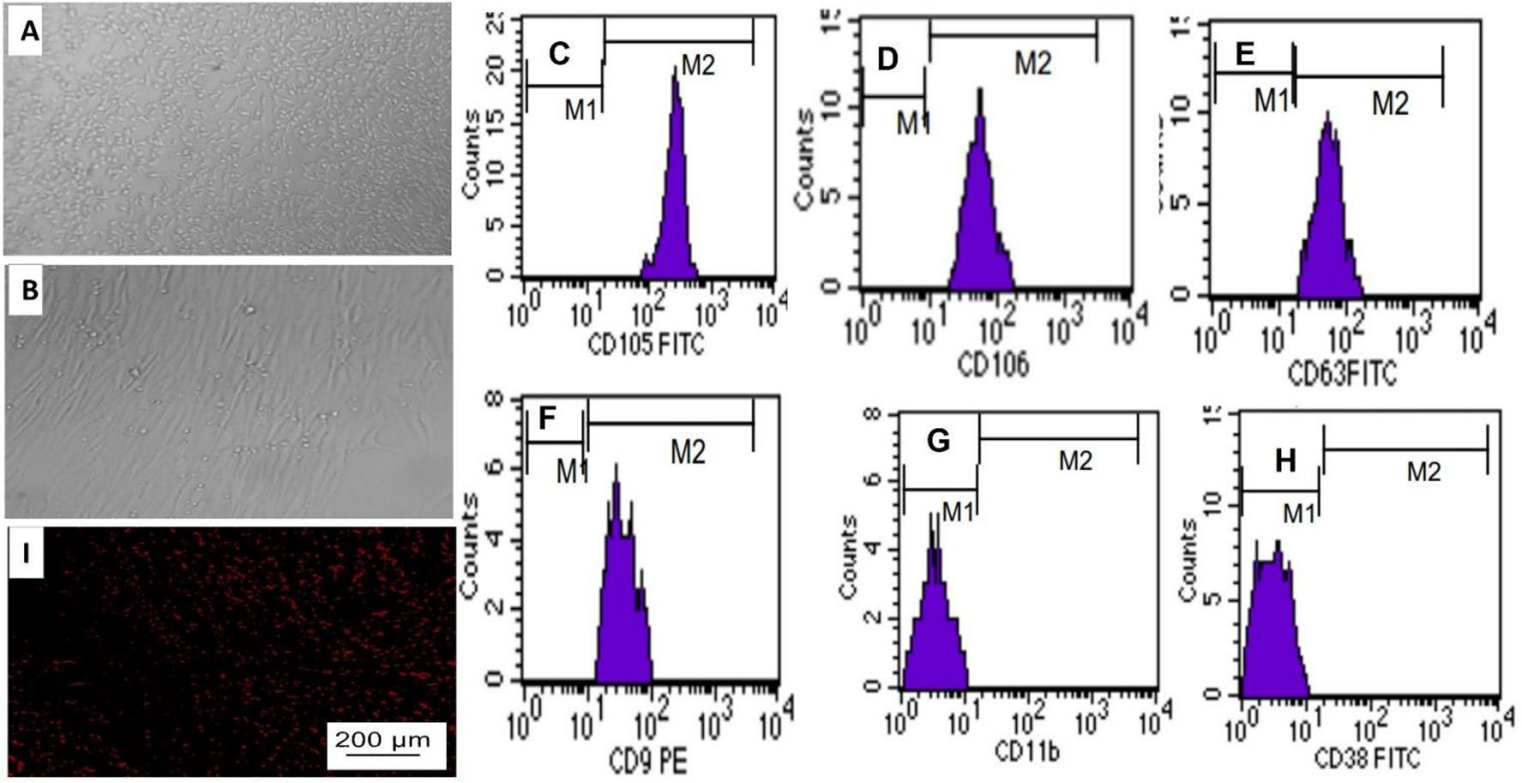
The adhesiveness and fusiform shape of MSc at day 14 were presented (**A**,**B**). Flowcytometry charts (M1; negative, M2; positive) showed MSc express CD105 (**C**), CD106 (**D**), CD63 (**E**) and CD9 (**F**) on cell surface however they do not express CD11 (**G**), or CD38 (**H**). (**I**) Representative fluorescence micrographs of cerebral cortex from toluene and breast milk-MSc-treated group following MSc dosing displaying the homing of MSc by PKH26 dye.

According to Patki, Kadam^17^, the capacity of the cultured breast milk MSc to attach to the base of the culture container with fibroblastic/spindle form was observed by the inverted image microscope (Fig. 1A,B).

In addition, flow cytometry was used for immunophenotyping of the MSc surface markers; 0.05% trypsin 0.02% EDTA in PBS was used to detach cells from the 3rd passage followed by suspension in DMEM and fixation in chilled 70% ethanol. Cells were then kept on ice with mouse anti-human antibodies against CD105, CD106, CD63, and CD9 (Becton Dickinson, San Diego, CA, USA) conjugated with fluorescein isothiocyanate (FITC)/phycoerythrin (PE) for 1 h at a dilution of 1:100. Flow cytometer laser (Becton Dickinson, New Jersey, NJ, USA) 488 nm with 10,000 gated cells was used to identify the cells. The data was then analyzed with BD Cellquest pro software, which included forward and side scattered gates. The separated MSc exhibit CD105, CD106, CD63, and CD9 on their cell surface but not CD11 or CD38 (Fig. 1 C-H). Before injection, The PKH26 Fluorescent Cell Linker Kit (Sigma–Aldrich) was used to label MSc for identification of MSc homing in cerebral tissue.

### Experimental design

#### Ethical approval

The experiment design was approved by the Institutional Animal Care and Use Committee (IACUC) of Zagazig University in Egypt (ZU-IACUC/3/F/110/2021) and conducted in accordance with ARRIVE guidelines.

Sixty male adult (12 ± 1 week) albino rats (220 ± 20 gm) were used in this study. Rats were obtained from the animal house, Faculty of Medicine, Zagazig University. The rats were kept in separate cages (2 rats per cage) and fed normal rat chow under regular laboratory and ambient conditions. The animals were handled according to the guidelines provided out in the National Institute of Health's Guide for the Care and Use of Laboratory Animals.

Each rat were then randomly allocated into one of three groups (Control group, Tol group, and Tol/MSc group; 20 rat each): control group was subdivided into vehicle subgroup were dermally exposed to olive oil only with the same method described in Tol group and MSc subgroup which received a single dose of breast milk-MSc (5*10^6^) for each rat; injected through the tail vein, after 14 days of the beginning dosing, toluene treated group (Tol group): an area about 10 cm^2^ on the dorsum of each rat was clipped free of hair 24 h. prior to dermal administration of toluene to avoid skin injury and washed with acetone, then a silicon ring (thickness × diameter; 2 mm × 34 mm) was glued to skin^18^ and cotton bad soaked in a previously prepared solution of 1% Tol in olive oil was applied inside the ring 5 h daily for 14 days^19^. A nylon mesh gauze was then glued to the silicone ring's surface, and a porous bandage was utilized to wrap the animal's trunk. After 5 h. of exposure, the bandage and gauze removed, and the skin washed with soap and water, toluene/MSc-treated group (Tol/MSc group): received toluene in the same regimen as Tol group and after the end of toluene administration the rats received a single dose of breast milk-MSc (5 × 10^6^) from individual donor for each rat; injected through the tail vein. Before transplantation, cells were re-suspended in 0.5 ml of DMEM (4.5 g/l glucose with L-glutamine, Lonza Bioproducts Walkersville, MD 21,793–0127 USA). The same volume of DMEM was injected into tail vein of the olive oil-treated and Tol-treated rats.

### Experimental animals’ general examination and neurological assessment

Three weeks after the mesenchymal stem cells transplantation, rats were weighted, observed for signs of general and local toxicity, and underwent neurological assessment. Following the method explained by Zin'kova, Gilerovich^20^, Six tests were used to measure neurological function; each was rated between 0 and 3, and each rat was assessed on an 18-point scale. These tests include spontaneous activity, symmetrical paw use during mobility, symmetrical use of front paws while they were the only animal support, symmetrical paw use to grab a mesh surface, reaction to body proprioception irritation, and reaction to vibrissa contact.

### Serum biochemical analysis

Three weeks after the stem cells injection, rats were anesthetized by thiopental intraperitoneal injection (50 mg/kg) and a blood sample from the retro-orbital plexus of each rat was collected by capillary glass tubes following the method explained by van Herck, Baumans^21^, The samples were allowed to spontaneously coagulate, followed by centrifugation at 3000 r.p.m for 10 min to separate sera which were then stored at − 20 °C for subsequent analysis of:

Glial fibrillary acidic protein (GFAP): GFAP ELISA Kit with cat no NS830 (Millipore, Temecula, CA) purchased from Sigma-Aldrich was utilized for the assessment. This kit uses a high-sensitivity Sandwich-ELISA principle. At 450 nm wavelength, the optical density (OD) was estimated spectrophotometrically. OD values were proportional to the rat GFAP concentration (ng/ml). Tumor necrosis factor-alpha (TNF-alpha): rat TNF-alpha ELISA Kit from MyBiosource (San Diego, California, United States) with cat no (MBS2507393) was used for its estimation. The Sandwich-ELISA principle was used in this ELISA kit. At a wavelength of 450 nm 2 nm, OD was measured spectrophotometrically where the values were correlated to the rat TNF- concentration. Nerve growth factor (NGF): was estimated using an ELISA kit from MyBiosource (San Diego, California, United States) with cat no (MBS355321) according to the manufacturer’s instruction. The amount of NGF in the sample (pg/ml) was proportionate to the intensity of the color developed which was estimated at 450 nm. Vascular endothelial growth factor (VEGF): the serum levels of VEGF in pg/ml were estimated via VEGF rat ELISA Kit from MyBiosource (San Diego, California, United States) with cat no (MBS724516) in accordance with the manufacturer’s instructions. The kit uses the competitive enzyme immunoassay method using an anti-VEGF antibody and a VEGF-HRP conjugate.

After obtaining blood samples, rats were sacrificed; cerebrum from each animal was carefully dissected and weighted, one half of the cerebral hemispheres was washed with ice-cold saline where a portion was homogenized using 0.1 M PBS at pH 7.4 to achieve a concentration of 10% w/v for the biochemical analysis (brain dopamine levels and oxidative stress parameters). Another portion was homogenized and stored at − 80 °C for later real time PCR analysis of surface markers of human MSc and gene expression analysis of Proliferator-Activated Receptor-Gamma (PPAR-ɣ), Nuclear factor-kappa B (NF-kB), and Interleukin-6 (IL-6). The other cerebral hemisphere was immersed immediately in buffered formalin 10% for 48 h to be handled for preparation of 5-μm-thick paraffin sections for fluorescent microscope examination, H&E and immunohistochemistry staining.

### Brain Biochemical analysis

Brain dopamine level was estimated using Rat dopamine, DA ELISA Kit from Cusabio Co., Wuhan, China with cat no (CSB-E08660r) following the manufacturer’s instruction. The kit using quantitative sandwich enzyme immunoassay technique. The intensity of the color generated at 450 nm was related to the amount of dopamine in the sample (ng/mg protein). Brain oxidative stress parameters: Lipid peroxidation products malondialdehyde (MDA): was estimated using a Biodiagnostic kit (Giza, Egypt with a cat. No. MD 2529) according to the instructions of the kit^22^. Nitric oxide: The NO level was determined by a Biodiagnostic kit where Vanadiumtrichloride was added to supernatants to reduce nitrate to nitrite following Miranda, Espey^23^. Glutathione peroxidase activity: The activity was measured in brain tissue (U/g protein) using Biodiagnostic kit following Paglia and Valentine^24^. Catalase activity (U/g protein): was estimated following the method of Aebi^25^. Superoxide dismutase (SOD) activity was measured (U/g protein) following Nishikimi, Appaji Rao^26^ using Biodiagnostic kit.

### Mesenchymal stem cells homing in cerebral tissue

Cerebral tissue was examined by fluorescent microscope for MSc treated groups. Moreover, determination of surface markers of human MSc using real time PCR for the following genes: Human GAPDH “*forward, 5′- GGTGAAGGTCGGAGTCAACG-3, reverse, 5′-CAAAGTTGTCATGGATGACC-3*”, Human CD105 “*forward, 5′-TCCTCCCAAGGACACTTGTA-3, reverse, 5′-CGCCTCATTGCTGATCATAC-3*”, and Human CD34 “*forward, 5′-GGAAGGATGCTGGTCCG-3, reverse, 5′-CTGGGGTAGCAGTACCGTTG-3*”.

### Quantitative reverse transcriptase-polymerase chain reaction (qRT-PCR) assessment of gene expression of PPAR-ɣ, NF-kB, and IL-6 in cerebral tissue

The total RNA was extracted from cerebral tissue homogenate by the miRNeasy extraction kit (QIAGEN; Valencia, CA) following the manufacturer´s recommendations. The NanoDrop spectrophotometer was used to determine the concentration and purity of the extracted RNA by estimating the OD at 260 and 280 nm and accepting A260/A280 at a ratio of 1.8–2.1. For reverse transcription and cDNA synthesis, Thermo Fisher Scientific's Applied BiosystemsTM high-capacity cDNA reverse transcription kit was employed. Expression levels of PPAR-ɣ, NF-kB, and IL-6 were evaluated by qRT-PCR using Mx3005P Real-Time PCR System (Agilent Stratagene, USA) consuming the primers in supplementary Table S1 and glyceraldehyde-3-phosphate dehydrogenase (GAPDH) as a housekeeping gene. The PCR mix used was 20 mL that contain 10 µl TOPreal™ qPCR 2X PreMIX (SYBR Green with low ROX), 1 µl of each primer (10 pmol/µl), 1 µl DNA template. The cycling parameters were primary denaturation at 95 °C for 12 min then 40 cycles of denaturation at 95 °C for 20 s, annealing at 60 °C for 30 s, and extension at 72 °C for 30 s. The obtained results were expressed as fold-change relative to the control group according to the 2^−ΔΔCT^ method^27^.

### Histopathological study

The paraffin sections of the frontal lobes of 10 rats/group were stained with hematoxylin and eosin (H&E) to demonstrate the histological structure according to Bancroft and Layton^28^, 10 fields from 3 sections from each rat were coded enabling blind examination & evaluation. Assessment Images were studied using imageJ software (ImageJ/Fiji 1.46r, https://imagej.nih.gov/ij/index.html). using 5 different non-overlapped horizontal sections from each animal, and the following parameters were measured: (1) the thickness of the frontal cortex; from each section, 20 perpendicular lines between the white and pia mater were quantified at 100 × magnification^29^. (2) Total number of neurons (pyramidal or granular) per field; at 400 × magnification^30^.

### Immunohistochemical examination of myelin basic protein (MBP) and high motility group box protein-1 (HMGB1)

For the immunohistochemical study, frontal lobes of the cerebrum from the other 10 rats/group were fixed overnight using 4% paraformaldehyde in PBS, dehydrated, and embedded in paraffin. Immunocytochemical marking was performed by monoclonal anti-MBP antibody and anti-HMGB1 antibody (Millipore Corporation Billerica, USA) on 5 μm thick cerebral sections. Deparaffinized sections were pre-incubated with citrate buffer and rinsed three times with 0.01 mol/L PBS. 0.3% hydrogen peroxide was used to treat the Section. After that, the sections were incubated for 1 h at 37 °C with 3% BSA to inhibit non-specific binding and then incubated with Anti-MBP (Millipore MAB382) and Anti-HMGB1 (Millipore MAB382) antibodies with 1:100 concentration. After rinsing with PBS, the sectioned tissue was treated for 10 min with the biotinylated secondary antibody. Following three times rinse with PBS, the section was washed with Streptavidin HRP. Immunoreactivity was visualized using DAB for 30 min. Consequently, slides were counterstained with Mayer's hematoxylin and fitted with a coverslip. Photomicrographs were taken with a light microscope (at × 400 magnification), 10 fields from 3 sections from each rat were coded to be examined and evaluated blindly. imageJ software (ImageJ/Fiji 1.46r, https://imagej.nih.gov/ij/index.html) was used for the calculation of the percentage of MBP immunoreactivity; 5 different non-overlapped sections from each animal were examined^31^. HMGB1-positive cells were defined as disorganized cells with dark brown stained cytoplasm. In each section, the positive cells were identified, numbered, and examined and were evaluated using imageJ software. The immunoreactivity of HMGB1 was used to compute the percentage of HMGB1 positive cells in each section; 5 different non-overlapped sections from each animal were examined^32^.

### Ethical approval

All animal experiments were carried out in accordance with relevant guidelines and regulations of the Institutional Animal Care and Use Committee (IACUC) of Zagazig University in Egypt (ZU-IACUC/3/F/110/2021).

## Results

There was no observed difference between vehicle-treated control subgroup and MSc subgroups as regard all the measures parameters.

### Effect of toluene ± MSc on general toxicity, body weight, brain weight, and neurological assessment

**Figure 2:**
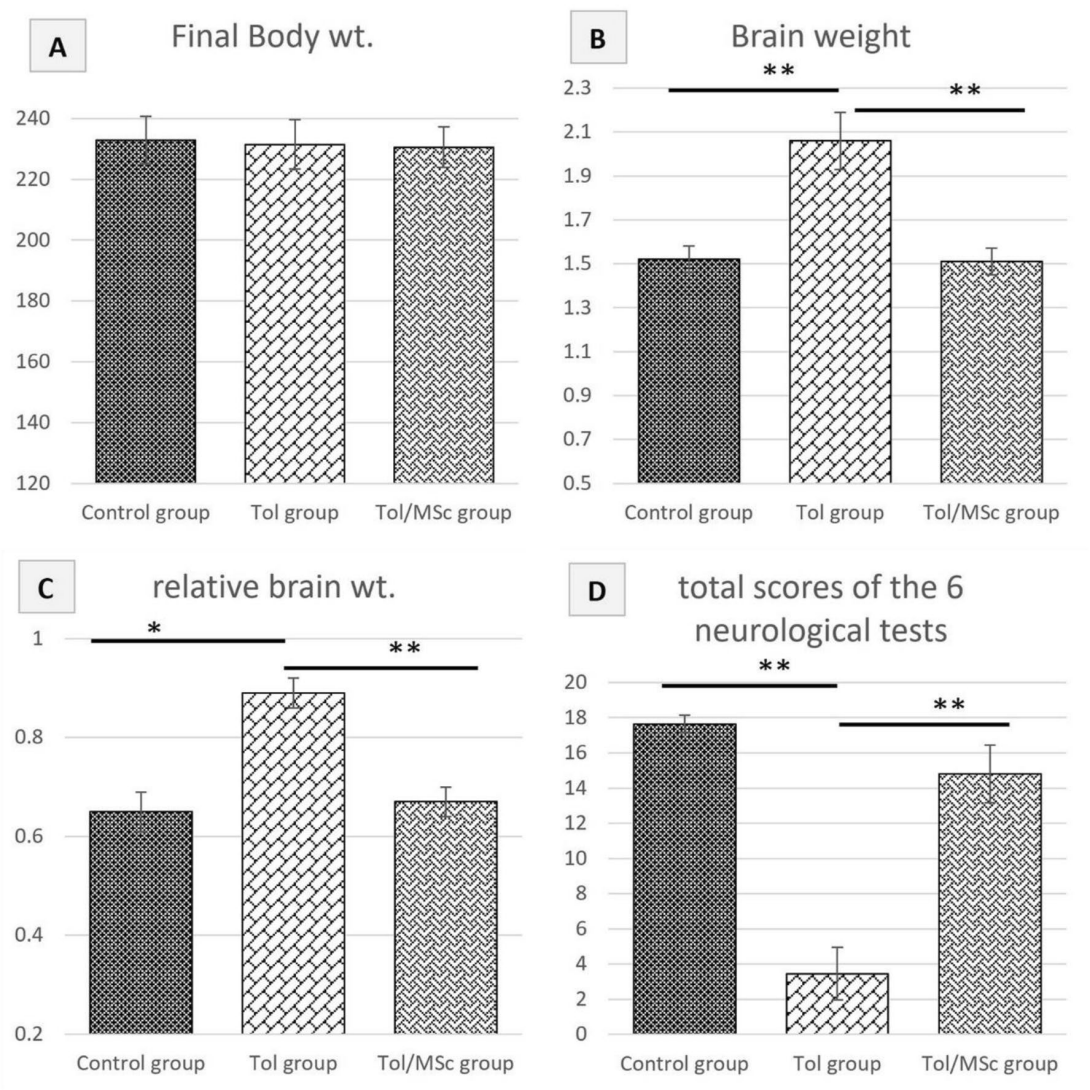
Charts (**A**) rat body weight, (**B**) rat brain weight, (**C**) relative brain weight “brain weight/body weight %” (**C**) total scores of the 6 neurological tests in different studied groups. *significant difference, **highly significant difference.

Minimal signs of dermal irritation in the form of redness were observed in Tol treated groups. However, no other signs of clinical toxicity, deaths or affection of body weight were detected in any of the groups. However, a significant increase in the brain weight in Tol group was noticed when compared with control and Tol/MSc groups (Fig. 2). The results of total scores of the 6 neurological tests showed significant decrease in Tol group vs control group, however, the neurological status recovered to the level of control animals in Tol/MSc group (Fig. 2).

### Cerebral homing of breast milk mesenchymal stem cells

Under florescent microscope, PKH26 tagged MSc appeared as bright spots in the cerebral tissue of the Tol and MSc treated group (Fig. 1I). moreover, PCR gene expression revealed positive expression of non-hematopoietic markers (human CD105) (1.09 ± 0.15) and negative expression of hematopoietic markers (human CD34) in the MSc treated groups. These results indicated successful homing of MSc in cerebral tissue.

### Effect of toluene ± MSc on oxidative and inflammatory markers

The current study's findings revealed that the Tol group had a significantly greater level of GFAP than the control group (p = 0.001), whereas the Tol/MSc group's levels were significantly lower than toluene (p = 0.001) but significantly higher than the control rats (p = 0.001) (Table 1).

**Table 1:** Statistical comparison of biochemical parameters. Glial fibrillary acidic protein (GFAP), tumor necrosis factor-alpha (TNF-α), nerve growth factor (NGF), vascular endothelial growth factor (VEGF) levels, tissue dopamine level, and tissue oxidative stress parameters (MDA, nitric oxide) among different studied groups.

	Control group (n20)	Tol group (n20)	Tol/MSc group (n20)	P value
GFAP (ng/ml)	8.27 ± 2.36	38.46 ± 9.03*	19.11 ± 3.82*^,^**	< 0.001
TNF-α (pg/ml)	92.61 ± 8.76	183.86 ± 21.43*	104.055 ± 12.52**	< 0.001
NGF (pg/ml)	661.67 ± 72.10	444.92 ± 90.25*	619.45 ± 90.38**	< 0.001
VEGF (pg/ml)	74.83 ± 8.63	73.39 ± 10.06	92.08 ± 9.78*^,^**	< 0.001
Dopamine level in brain (ng/mg protein)	0.22 ± 0.31	0.16 ± 0.017*	0.18 ± 0.020*^,^**	< 0.001
MDA (nmol/g)	16.71 ± 2.32	34.35 ± 5.31*	20.80 ± 3.69*^,^**	< 0.001
NO (nmol/g)	17.25 ± 4.52	47.35 ± 11.20*	23.51 ± 7.98**	< 0.001
Glutathione peroxidase (U/g)	30.83 ± 3.02	15.50 ± 3.59*	24.63 ± 3.88*^,^**	< 0.001
Catalase (U/g protein)	3.03 ± 0.185	1.16 ± 0.261*	2.42 ± 0.423*^,^**	< 0.001
SOD (U/g)	2.11 ± 0.305	1.058 ± 0.253*	1.53 ± 0.423*^,^**	< 0.001

One-way ANOVA, and Tukey HSD Post-hoc Test, P > 0.05: no significant differences, P < 0.05: significant differences.

*Significant vs control group.

**Significant vs Tol group.

Table 1 also showed that toluene administration caused a significant elevation in the TNF-α compared to the control rats (p = 0.001) and the concentrations attenuated significantly by the concurrent breast milk-MSc administration compared to toluene treatment (p = 0.001) with non-significance from control (p = 0.53).

As shown in Table 1, the current study revealed a significant decrease in NGF level in toluene treated group upon comparing to the control rats (p < 0.01) and the concentrations significantly increased in Tol-MSc treated group (p < 0.01) with non-significant variation from the control (p = 0.264).

Table 1 revealed a non-significant difference in VEGF serum levels between the control and Tol-treated groups (p = 0.87), while the levels were significantly greater in Tol-MSc than control (p = 0.001), and Tol-treated group (p = 0.001).

The brain dopamine level in Table 1 showed a significant decrease in the toluene-exposed rats compared to the control rats (p = 0.001), while the level in the toluene and breast milk MSc was significantly higher in Tol-treated rats (p = 0.008) and significantly lesser than the control rats (p = 0.001).

As regard the oxidate stress parameters, the level of MDA was significantly greater in the Tol-exposed rats compared to the control rats (p = 0.001), while levels decreased significantly with Tol-MSc treatment comparing to Tol group (p = 0.001) and significantly greater than control (p = 0.005). There was a significant elevation in NO in Tol-group in relation to the control levels (p = 0.001), while the values in Tol/MSc group decreased significantly than the Tol group (p = 0.001) and non-significantly different from the control (p = 0.054) (Table 1).

As shown in (Table 1), the levels of the antioxidant enzyme SOD, CAT, GPx decreased significantly in the Tol-group in relation to the control group (p = 0.001), and the level increased significantly with combined toluene and breast milk MSc treatment (p = 0.001) but still lower than control values (p = 0.001).

### PPAR-ɣ, NF-kB, and IL-6 gene expression in cerebral tissue

Table 2 showed that toluene treatment in the Tol group induced a significant reduction in PPAR-ɣ gene expression in relation to the control group (p = 0.001). The co-treatment with breast milk-MSc in Tol/MSc group resulted in a significant elevation in PPAR-ɣ gene expression in relation to the Tol-group (p = 0.001) with a non-significant change from control (p = 0.775) and there was a strong positive correlation between PPAR-ɣ gene expression and levels of VEGF (r = 0.8285 and p = 0.001).

**Table 2:** Statistical comparison of gene expression of peroxisome Proliferator-Activated Receptor-Gamma (PPAR-ɣ), Nuclear Factor kappa B (NF-kB), and Interleukin-6 (IL-6).

	Control group (n20)	Tol group (n20)	Tol/MSc group (n20)	P value
PPAR-ɣ	1.0179 ± 0.083	0.344 ± 0.117*	1.0375 ± 0.082**	< 0.001
NF-kB	1.0232 ± 0.117	5.626 ± 1.816*	1.2917 ± 0.3639**	< 0.001
IL-6	1.0458 ± 0.322	4.1645 ± 0.533*	1.8825 ± 0.3626*^,^**	< 0.001

One-way ANOVA, and Tukey HSD Post-hoc Test, P > 0.05: no significant differences, P < 0.05: significant differences.

*Significant vs control group.

**Significant vs Tol group.

Comparing the Tol-group to the control group, there was a significant elevation in the gene expression of NF-kB (p = 0.001). The levels reduced in the toluene and breast milk MSc in relation to the Tol group (p = 0.001) and they did not differ significantly from the control group. (p = 0.692). There was a strong positive correlation between serum levels of TNF-α and the NF-kB gene expression (r = 0.853, p = 0.001).

Upon comparing the Tol group to the control group, there was a substantial increase in IL-6 gene expression (p = 0.001). The values in the Tol/MSc group were lesser than in the toluene group (p = 0.001), but still significantly above than the control group (p = 0.001).

### Effect of toluene ± MSc on histological structure of cerebral tissue

**Figure 3:**
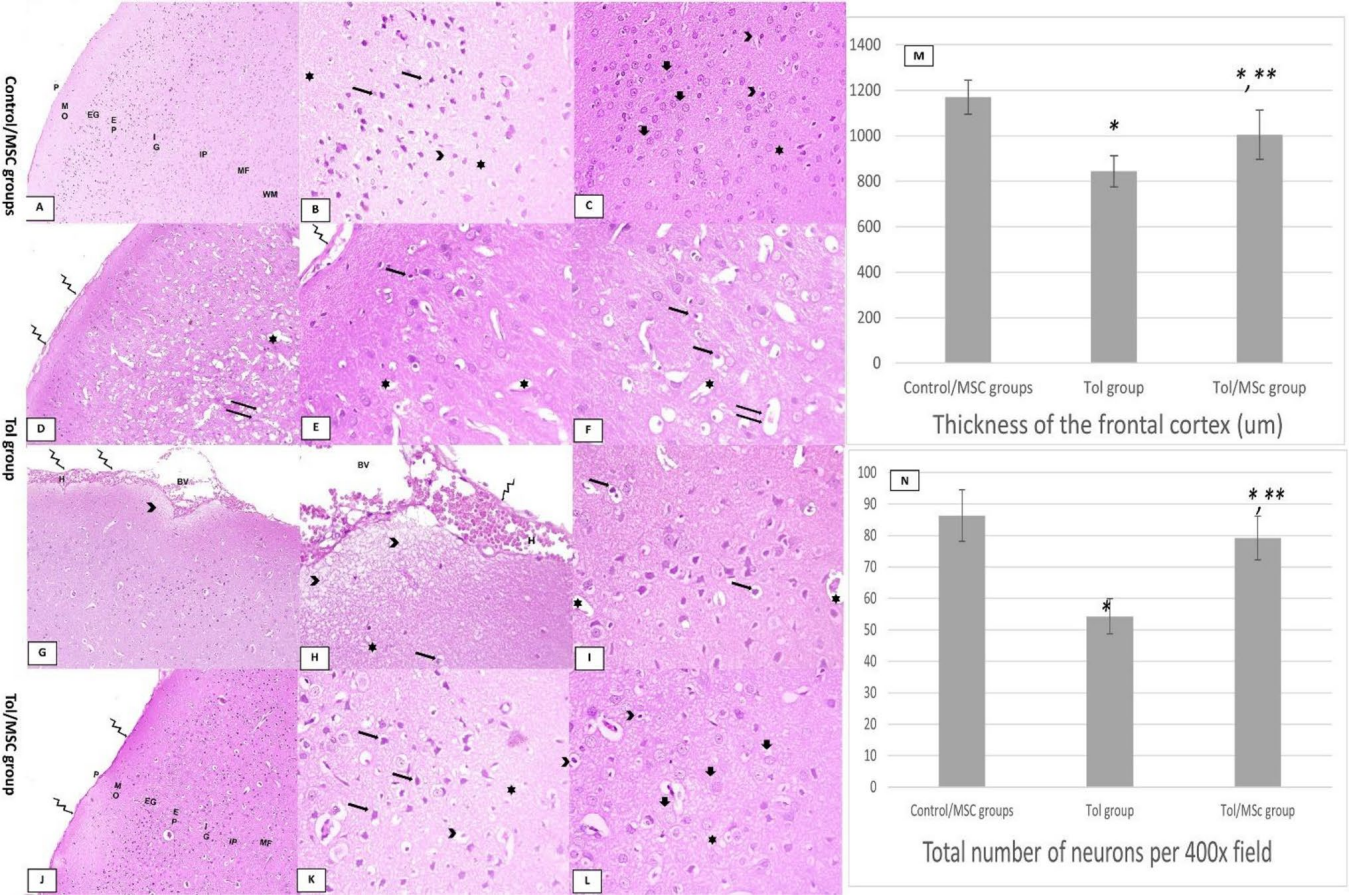
H&E Photomicrographs of the cerebral tissue. (**A**) A 100 × H&E micrograph of control and MSC groups presenting the layers of the cerebral cortex; outer molecular layer (MO), external granular layer (EG), external pyramidal layer (EP), internal granular layer (IG), internal pyramidal layer (IP), and multiform layer (MF), in addition to white matter (WM), pia matter thin and normally attached to the cortex (p), (**B**,**C**) a 400 × H&E micrographs exhibiting the pyramidal cells characterized by basophilic cytoplasm and apical processes (arrows), the granular cells showing vesicular and prominent nucleoli (thick arrows), the glia cells showing dense small nuclei (arrowheads), and a pink neuropil homogenous background (star). Photomicrographs taken from toluene-treated rat showing (**D**,**G** 100 × H&E, **E**,**F**,**H**,**I** 400 × H&E); meninges showing signs of inflammation with inflammatory cell infiltration and exudation (zigzag arrows) with congested, dilatated cortical blood vessels (BV) with extravasation and hemorrhage (**H**), neuropil background lost its normal homogeneity with the appearance of vacuolations (stars) and hydropic changes (arrowheads), neurons containing pyknotic hyperchromatic nuclei inside vacuolated cytoplasm (arrows), also, the white matter appeared vacuolated (double arrows). Photomicrographs taken from the toluene and breast milk MSc-treated group (**J**) a 100 × H&E micrograph of showing the different layers of the cerebral cortex; (**K**,**L**) 400 × H&E micrographs showing the recovery of the basic histological construction, pyramidal cells (arrows), granular cells (thick arrows) with scattered pyknotic nuclei (arrowheads), white matter and neuropil was with fewer vacuolations (stars). Meninges exhibited normal architecture to the cerebral surface (zigzag arrows). (**M**,**N**) charts showing quantitative assessment of the cortical thickness and total no. of neuron/ filed. Data were expressed as mean ± SD, *significant vs control group, **significant vs toluene group.

Hematoxylin and eosin stained cerebral tissues from control subgroups exhibited normal histological pattern regarding the cerebral cortex and white matter, the cortex was composed of six cell layers; molecular layer, outer granular layer, outer pyramidal layer, inner granular layer, inner pyramidal layer, and multiform layer, in addition, the white matter showed normal homogenous structure with no vacuolations (Fig. 3A). Cortical layers consisted of different populations of cells with scattered small dark glial cells in a pink neuropil homogenous background. The pyramidal cells characterized by basophilic cytoplasm and apical dendrites (Fig. 3B), the granular cells were characterized by vesicular nuclei and prominent nucleoli (Fig. 3C). Conversely, although 3 weeks since last toluene dose, the Tol group revealed variation of the characteristic cortical composition which appeared as cellular degeneration of cortical cells (pyknotic hyperchromatic nuclei within vacuolated cytoplasm), moreover, white matter and neuropil background lost their normal homogeneity with the appearance of vacuolations and hydropic changes, meninges showing signs of inflammation with inflammatory cell infiltration and exudation, cortical blood vessels were congested, dilated with extravasation and hemorrhage (Fig. 3D–I). In Tol/MSc group, cerebral cortical tissues maintained their normal proportions and architectures. There were several neurons with vesicular nuclei and prominent nucleoli and some neurons with darkly stained nuclei. Additionally, white matter and neuropil homogenous background were with fewer vacuolations and less hydropic changes. Meninges were normally attached to the cerebral surface (Fig. 3J–L).

Adding to the pathological lesions of the cortical cells, the thickness of the frontal cortex and the total number of neurons of the Tol group were significantly lower than that of the control (P < 0.0001). Mesenchymal stem cells administration led to repair of these histopathological changes (Fig. 3 M, N, Table 3).

**Table 3:** Statistical comparison of the morphometric analysis for H&E histological examination (thickness of the frontal cortex, total numbers of neurons) and the area percentage (area %) of MPB immunoreaction and HMGB1 positive cells among different studied.

	Control group (n20)	Tol group (n20)	Tol/MSc group (n20)	P value
Thickness of the frontal cortex (um)	1169.73 ± 74.66	843.5 ± 69.75*	1005.12 ± 108*^,^**	< 0.001
Total number of neurons/400 × field	86.38 ± 8.2	54.30 ± 5.6*	79.20 ± 6.9*^,^**	< 0.001
% of MBP immunoreactivity	66.7 ± 6.5	41.6 ± 5.2*	57.6 ± 4.4*^,^**	< 0.001
% of HMGB1 positive cells	9.5 ± 2.1	50.6 ± 11*	23.3 ± 4.8*^,^**	< 0.001

One-way ANOVA, and Tukey HSD Post-hoc Test, P > 0.05: no significant differences, P < 0.05: significant differences.

*Significant vs control group.

**Significant vs Tol group.

### Immunohistochemical examination of cerebral tissue

**Figure 4:**
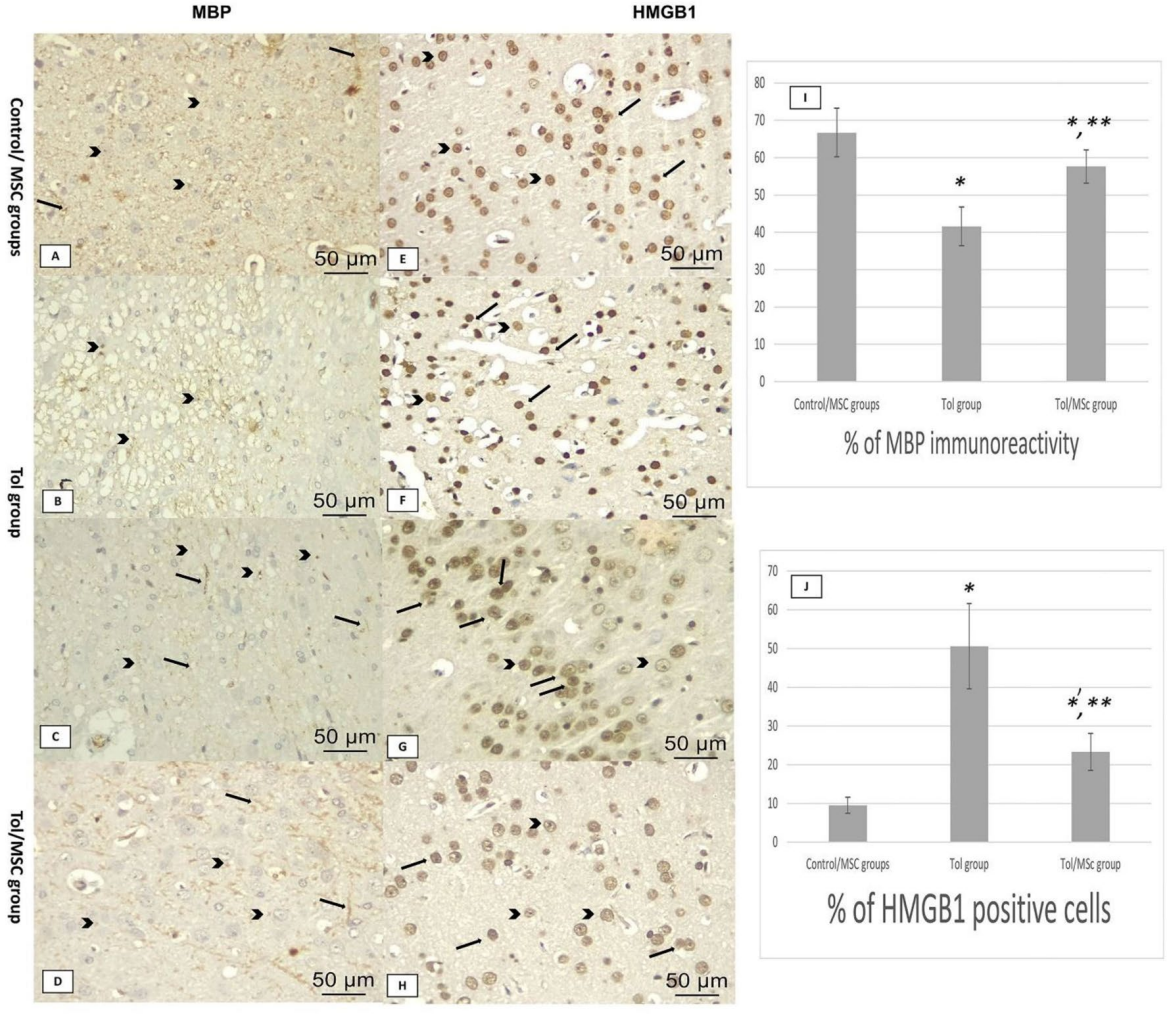
Immunohistochemical staining for anti-MBP, anti- HMGB1; 400 × magnification. Anti-MBP (**A** control group; **B**,**C** toluene-treated group; **D** toluene and breast milk MSc-treated group) stained myelin fibers appear as rounded droplets if transverse cuts (arrows heads) and fine lines if longitudinal cuts (arrows) showing b: decreased density of myelin. (**I**) charts showing quantitative analysis of the percentage of MBP immunoreactivity. Anti- HMGB1 (**E** control group; **F**,**G** toluene-treated group; **H**: toluene and breast milk MSc-treated group) Anti-HMGB1 stained normal cell bodies appear intact with a dark brown nucleus and faint cytoplasm (arrows heads). HMGB1-positive cells appeared disarranged with dark brown stained cytoplasm (arrows). (**J**) charts showing quantitative analysis of the percentage of HMGB1 positive cells in each group. Data were represented as mean ± SD, *Significant vs control group, **significant vs toluene group.

Most immunohistochemical stained myelin fibers with anti-MBP looked rounded droplets (transverse cuts), also some immunohistochemically stained myelin fibers looked like fine lines (longitudinal cuts). Calculation of percentage of MBP immunoreactivity revealed that it was significantly lower in the Tol group compared to the control group, indicating that toluene induced loss of myelin sheath in cerebral tissue. However, therapy with breast milk MSc in the third group showed great improvement of MBP immunoreactivity with a significant difference compared to both control and toluene-treated groups (Fig. 4A–D,I, Table 3).

Anti-HMGB1 stained normal cell bodies appeared with a dark brown nucleus and faint cytoplasm with intact appearance, while HMGB1-positive cells appeared disarranged with dark brown stained cytoplasm. Calculation of the percentage of HMGB1 positive cells in each group revealed a highly significant elevation in the toluene-exposed rats indicating that toluene induced neuroinflammation in cerebral tissue. However, therapy with mesenchymal stem cells in group III showed great improvement of HMGB1 immunoreactivity with a significant difference compared to both control and toluene-exposed rats (Fig. 4E–H,J, Table 3).

## Discussion

The current work proved that toluene induced cerebral injury manifested by significant decrease in scoring of the neurological tests with increase in the brain weight. Also, there was evident significant increase in GFAP. GFAP was a cytoskeletal protein that served as a hallmark of atypical astrocyte activation and proliferation caused by neuronal injury^33^. GFAP was increased after CNS injuries, neurodegeneration, stroke^34^, and exposure to various groups of toxic chemicals that include piperidine alkaloids, mercury and aluminum chloride, kainic acid, toluene, ethanol, dibutyryl-cAMP, and trimethyltin^35^. Besides, the current study revealed that toluene induced a significant decrease in brain dopamine levels. There was a controversy in the previous reports regarding the toluene effect on dopamine^36,37^; reported that toluene contact increases dopamine concentrations in prefrontal cortex, the striatum, and ventral tegmental area respectively, however^38^, reported that toluene decreases the amount of striatal dopamine metabolites. This variability was mostly related to the duration of toluene exposure with a tendency to decreased dopamine in longer exposures^39^. It was documented that dopamine balance was essential for motor coordination and mental wellbeing and its decrease was associated with anxiety-like behavior^40^. In addition, toluene caused alteration of the normal cerebral structure and cellular degeneration with a considerable reduction in the total number of neurons and thickness of the frontal cortex, meninges showing signs of inflammation with inflammatory cell infiltration and exudation, this result in accordance with^41,42^, who reported similar histopathological changes of toluene on rabbit brain. In addition, the toluene-induced cerebral injury was manifested in the current work by the loss of myelin sheath in cerebral tissue evidenced by the significant reduction in MBP immunoreactivity. MBP was the second most prevalent myelin sheath protein in the central nervous system (CNS), accounting for 30% of myelin protein concentration in the white matter^43^. Chemical toxicity, traumatic brain damage, and demyelinating illness were all examples of CNS stressors that can cause myelin degeneration^44^. Demyelination induces detrimental inflammation^45^, and cell death^46^. Chronic toluene abuse produces a devastating neurological disorder, of which dementia is the most disabling component^3^. Moreover Feldman, Ratner^47^ reported impaired short-term memory function, changes in affect in his early forties and persistent cognitive deficits in a 57-year-old painter who work as a painter. Magnetic resonance imaging revealed a symmetrical global volume reduction including more white matter than grey matter. The results in this patient are consistent with chronic toxic encephalopathy, which may be distinguished from Alzheimer's disease, multi-infarct (vascular) dementia, and alcoholic dementia. The findings in this case are supported by previous accounts in the literature of lasting neurobehavioral consequences linked with prolonged exposure to organic solvents.

The current work showed that the toluene-induced cerebral injury was multifactorial; toluene included an inflammatory reaction in tissues proved by the significant increase in TNF-α. Toluene exposure was reported to be associated with systemic inflammation^48^, and also with organ inflammation like the airway passages^49,50^ and nervous system^51^. The current work demonstrated an increased nuclear factor-kappa B (NF-κB) gene expression that was positively correlated to the serum levels of TNF-α. Liu, Zhang^52^ reported that NF-kB was activated by several stimuli including TNF-α, interferon-gamma (IFN-γ), interleukins, bacterial and viral infection. The inflammatory reaction initiated by the inflammatory cytokines as TNF-α promote the accumulation of inflammatory cells that express iNOS and yield vast quantities of NO^53^; which was found to be elevated in the current study. Also, the activation of NF-κB highly expresses iNOS which continuously produces NO^54^. Another finding that proved the toluene-induced inflammation in cerebral tissue was the significant increase in HMGB1 immunoreactivity and the IL-6 gene expression. According to Shimizu, Kouzaki^55^, TNF-α up regulated the generation and secretion of IL-6 via stimulating the production of HMGB1. An increase in HMGB1 expression after toluene exposure was reported in many organs like the lung and bronchi^50^. The pro-inflammatory cytokine IL-6 was present in the CNS and was activated during the acute inflammatory response^56^. This pro-inflammatory pathway was shown to mediate neurodegeneration in mice^57^. In addition, in traumatic brain injury, IL-6 was a well-established biomarker for confirming the presence and severity of brain lesions as well as predicting the probable neurological outcome^58^. Moreover, there was an evident elevation in the oxidation product MDA associated with a reduction in the antioxidant parameters GPx, catalase, and superoxide dismutase. In accordance with these results, Kamel and Shehata^59^ showed that toluene toxicity was caused by oxidative stress and an imbalance in the GSH/GSSG ratio, which causes chronic inflammation and apoptosis.

The current work proved a significant decrease in the neurotrophic nerve growth factor in the toluene-treated group in accordance with Demır, Cicek^41^ who found decreased levels of NGF in hippocampal neurons of the rabbit brain. Nerve growth factor (NGF) was the first neurotrophic factor discovered and studied, and it was known to be important in the development and persistence of many neurons in the peripheral and central nervous systems. It furthermore aids in the recovery of nerve cells following ischemia, surgical, or chemical damage^60^. The concurrent MSc treatment significantly increased the NGF. Previous reports demonstrated that Neurotrophic factors such as nerve growth factor (NGF), neurotrophin-3 (NT-3), glial cell line-derived neurotrophic factor (GDNF), fibroblast growth factor-2, and insulin-like growth factor type 1 have been found to be released by human MSc^61,62^.

The current work proved that there was a significant decrease in PPAR-ɣ expression by toluene treatment which was reversed by the administration of breast milk MSc. This observation can be explained by the capacity of NGF to promote the transcriptional activity of PPAR-ɣ^63^. In addition, there was an evidenced increase in VEGF level induced by breast milk MSc with a positive correlation between the expression of PPAR-γ and serum levels of VEGF. According to Li, Li^64^, NGF increases angiogenesis in the rat brain following cerebral infarction by enhancing the in vitro and in vivo capillary-like tube development in microvascular endothelial cells. Chen, Zhang^65^ showed that PPAR-γ was considered an activator of the PI3K/Akt signaling. Di, Gao^66^ revealed that stimulation of the PI3K/AKT signaling increases VEGF mRNA expression and the VEGF level.

In addition, the decreased expression of PPAR-γ induced by toluene treatment in the current work was an interesting and crucial finding since it can explain the underexplored effect of toluene on energy homeostasis reported in previous studies^67,68^. PPAR-γ helps the maintenance of energetic homeostasis possibly by promoting mitochondrial activity^69^. In addition, the proven enhancement of PPAR-γ gene expression with the concurrent breast milk MSc treatment can explain the improved myelination of the nerve cells with the significant increase in MBP immunoreactivity. In accordance, De Nuccio, Bernardo^70^ suggested that PPAR-γ agonists promotes myelination via many mechanisms, including enhancing the functions of the mitochondria and affecting oscillatory Ca (2 +) waves. In addition, PPAR-ɣ also induces reduction of inflammation, inhibition of oxidative stress, decrease of pro-apoptotic factors^53^.

Stem cells have been proposed as an increasingly attractive approach for repair of damaged nervous system. So, we aimed to evaluate the ability of breast milk mesenchymal stem cells (MSc) to ameliorate toluene-induced encephalopathy. Furthermore, to minimized MSc heterogeneity, in Tol/MSc group; each rat received breast milk MSc from single donor. As, adult tissues generated MSc were very diverse. These MSc produced from various individuals/donors frequently exhibit batch-to-batch differences, stem cell senescence, and proliferative potency, all of which impair the accuracy of MSc research. As a result, MSc can be produced from the same parental pluripotent stem cell (PSC), which overcomes several of the shortcomings of adult MSc, such as more homogenous MSc quality with reduced batch-to-batch fluctuation, stem cell senescence, and restricted proliferative ability^71–73^.

The current work also proved that concurrent administration of breast milk MSc with toluene attenuate significantly the oxidative stress parameters and enhance the measured antioxidants supporting the previously reported findings of Stavely and Nurgali^74^ where they suggested that MSc have antioxidant activities in variable in vitro and in vivo animal models of diseases explaining their cytoprotective and anti-inflammatory properties. The antioxidant properties were mediated through enhancing the endogenous antioxidant defense and direct free radicals scavenging, immunomodulation through suppression of reactive oxygen species, modification of mitochondrial bioenergetics, and donating efficient mitochondria to the damaged cells. Moreover, Staff, Jones^75^ reported that Mesenchymal stromal cells are multipotent cells that are being used to treat a variety of medical conditions. Over the past decade, there has been considerable excitement about using MSc to treat neurodegenerative diseases such as amyotrophic lateral sclerosis, multiple system atrophy, Parkinson disease, and Alzheimer disease. Continued rigorous and controlled studies of MSc therapies are critical to establish efficacy and protect patients from possible untoward effects.

## Conclusion

Toluene was believed to be the most prevalent hydrocarbon in the environment owing to its use in a wide variety of commercial and household products. For both acute and chronic toluene poisoning in humans and animals, the central nervous system was the major target organ. Stem cell-based regenerative medicine has attracted attention for the treatment of different neurological pathologies. The current study describes for the first time the ability mesenchymal stem cells to treat toluene-induced encephalopathy in adult male albino rats. Upon comparing toluene treated rats to controls, the toluene administration resulted in cerebral injury proved biochemically, histologically and immunohistochemically. The toluene induced CNS injury mainly through inflammatory and oxidative stresses. The breast milk MSc greatly improved the toluene-induced encephalopathy. The study points to the role of Peroxisome Proliferator-Activated Receptor Gamma (PPAR-ɣ) signaling pathway in the amelioration of the toluene-induced cerebral injury.

## Supplementary Information

Supplementary Tables.

## Data Availability

The data sets used and analyzed in this current study were available from the corresponding author upon reasonable request.

## References

[1] Tas U (2011). Hepatotoxic activity of toluene inhalation and protective role of melatonin. Toxicol. Ind. Health.

[2] Lim SK (2014). Risk assessment of volatile organic compounds benzene, toluene, ethylbenzene, and xylene (BTEX) in consumer products. J. Toxicol. Environ. Health A.

[3] Filley CM, Halliday W, Kleinschmidt-Demasters BK (2004). The effects of toluene on the central nervous system. J. Neuropathol. Exp. Neurol..

[4] Hannigan JH, Bowen SE (2010). Reproductive toxicology and teratology of abused toluene. Syst. Biol. Reprod. Med..

[5] Malaguarnera G (2012). Toxic hepatitis in occupational exposure to solvents. World J. Gastroenterol..

[6] Camara-Lemarroy CR (2015). Acute toluene intoxication–clinical presentation, management and prognosis: A prospective observational study. BMC Emerg. Med..

[7] Meydan S (2013). The protective effects of caffeic acid phenethyl ester against toluene-induced nephrotoxicity in rats. Toxicol. Ind. Health.

[8] Cruz, S.L., M.T. Rivera-García, & J.J. Woodward. Review of toluene action: Clinical evidence, animal studies and molecular targets. *J. Drug Alcohol Res*. **3** (2014). 10.4303/jdar/235840 PMC4211428 25360325

[9] Zeng F (2014). Toluene-induced leukoencephalopathy with characteristic magnetic resonance imaging findings. Neuroimmunol. Neuroinflam..

[10] Filley CM (2013). Toluene abuse and white matter: A model of toxic leukoencephalopathy. Psychiatr. Clin. North Am..

[11] Kassis I (2013). Mesenchymal stem cells in neurological diseases. J. Clin. Invest..

[12] Andrzejewska A (2021). Mesenchymal stem cells for neurological disorders. Adv. Sci..

[13] Fan Y (2010). Endothelial progenitor cell transplantation improves long-term stroke outcome in mice. Ann. Neurol..

[14] Park H-J (2011). neuroprotective effect of human mesenchymal stem cells in an animal model of double toxin-induced multiple system atrophy parkinsonism. Cell Transplant..

[15] Ali E, Ahmed-Farid O, Osman A (2017). Bone marrow-derived mesenchymal stem cells ameliorate sodium nitrite-induced hypoxic brain injury in a rat model..

[16] Streicher RP (2000). Determination of airborne isocyanate exposure: considerations in method selection. AIHAJ.

[17] Patki S (2010). Human breast milk is a rich source of multipotent mesenchymal stem cells. Hum. Cell.

[18] Hoffmann, H.D., et al., Dermal uptake and excretion of 14C-toluene diisocyante (TDI) and 14C-methylene diphenyl diisocyanate (MDI) in male rats. Clinical signs and histopathology following dermal exposure of male rats to TDI. *Toxicol. Lett.* **199**(3), 364–371 (2010). 10.1016/j.toxlet.2010.09.021 20933064

[19] Yeh H-J (2008). Urinary excretion of toluene diisocyanates in rats following dermal exposure. J. Appl. Toxicol. JAT.

[20] Zin'kova NN (2007). Mesenchymal stem cells-based therapy of brain ischemic stroke in rat. Tsitologiia.

[21] van Herck H (1998). Orbital sinus blood sampling in rats as performed by different animal technicians: The influence of technique and expertise. Lab. Anim..

[22] Ohkawa H, Ohishi N, Yagi K (1979). Assay for lipid peroxides in animal tissues by thiobarbituric acid reaction. Anal. Biochem..

[23] Miranda KM, Espey MG, Wink DA (2001). A rapid, simple spectrophotometric method for simultaneous detection of nitrate and nitrite. Nitric Oxide.

[24] Paglia DE, Valentine WN (1967). Studies on the quantitative and qualitative characterization of erythrocyte glutathione peroxidase. J. Lab. Clin. Med..

[25] Aebi H (1984). [13] Catalase in vitro. Methods in Enzymology.

[26] Nishikimi, M., Appaji Rao, N., Yagi, K. The occurrence of superoxide anion in the reaction of reduced phenazine methosulfate and molecular oxygen. *Biochem. Biophys. Res. Commun.* **46**(2), 849–854 (1972). 10.1016/s0006-291x(72)80218-3 4400444

[27] Livak KJ, Schmittgen TD (2001). Analysis of relative gene expression data using real-time quantitative PCR and the 2−ΔΔCT method. Methods.

[28] Bancroft JD, Layton C, Suvarna SK, Layton C, Bancroft JD (2013). 10 - The hematoxylins and eosin. Bancroft's Theory and Practice of Histological Techniques (Seventh Edition).

[29] Bahey NG, Elaziz HOA, Gadalla KKES (2015). Toxic effect of aflatoxin B1 and the role of recovery on the rat cerebral cortex and hippocampus. Tissue Cell.

[30] Abdel-kareem, R., & Domouky, A. Role of Β-carotene against toxic effect of titanium dioxide nanoparticles on cerebral cortex of adult albino rat: Histological and biochemical approach.* J. Egypt. J. Histol.* **43**(2), 441–454 (2020).

[31] Hassen, E.Z., et al., The effect of long term administration of aspartame on the sciatic nerve of adult male albino rats and the possible therapeutic role of ozone (histological and biochemical study). *J Egypt. J. Histol.* **42**(1), 191–201 (2019).

[32] Tian J (2015). The effect of HMGB1 on sub-toxic chlorpyrifos exposure-induced neuroinflammation in amygdala of neonatal rats. Toxicology.

[33] Colangelo AM, Alberghina L, Papa M (2014). Astrogliosis as a therapeutic target for neurodegenerative diseases. Neurosci. Lett..

[34] Yang Z, Wang KK (2015). Glial fibrillary acidic protein: from intermediate filament assembly and gliosis to neurobiomarker. Trends Neurosci..

[35] Pitanga, B., et al., The role of astrocytes in metabolism and neurotoxicity of the pyrrolizidine alkaloid monocrotaline, the main toxin of crotalaria retusa. **3** (2012). 10.3389/fphar.2012.00144 PMC3411086 22876233

[36] Gerasimov MR (2002). Toluene inhalation produces regionally specific changes in extracellular dopamine. Drug Alcohol Depend..

[37] Riegel AC (2007). The abused inhalant toluene increases dopamine release in the nucleus accumbens by directly stimulating ventral tegmental area neurons. Neuropsychopharmacology.

[38] Kim J (2020). Toluene Inhalation Causes Early Anxiety and Delayed Depression with Regulation of Dopamine Turnover, 5-HT(1A) Receptor, and Adult Neurogenesis in Mice. Biomol Ther (Seoul).

[39] Apawu AK, Mathews TA, Bowen SE (2015). Striatal dopamine dynamics in mice following acute and repeated toluene exposure. Psychopharmacology.

[40] Zarrindast MR, Khakpai F (2015). The modulatory role of dopamine in anxiety-like behavior. Arch. Iran. Med..

[41] Demır M (2017). Effects of acute toluene toxicity on different regions of rabbit brain. Anal. Cell. Pathol..

[42] Salam O (2021). Effect of piracetam on brain oxidative stress and tissue damage following toluene exposure in rats. Int. J. Halal Res..

[43] Boggs JM (2006). Myelin basic protein: A multifunctional protein. Cell. Mol. Life Sci. CMLS.

[44] Zhang J (2014). Myelin basic protein induces neuron-specific toxicity by directly damaging the neuronal plasma membrane. PLoS ONE.

[45] Sun X (2010). Myelin activates FAK/Akt/NF-κB pathways and provokes CR3-dependent inflammatory response in murine system. PLoS ONE.

[46] Hagemeier K (2013). Puma, but not noxa is essential for oligodendroglial cell death. Glia.

[47] Feldman RG, Ratner MH, Ptak T (1999). Chronic toxic encephalopathy in a painter exposed to mixed solvents. Environ. Health Perspect..

[48] Werder EJ (2020). Blood BTEXS and heavy metal levels are associated with liver injury and systemic inflammation in Gulf states residents. Food Chem. Toxicol..

[49] Zhuang J (2020). Bronchial epithelial pyroptosis promotes airway inflammation in a murine model of toluene diisocyanate-induced asthma. Biomed. Pharmacother..

[50] Jiao B (2021). Toluene diisocyanate-induced inflammation and airway remodeling involves autophagy in human bronchial epithelial cells. Toxicol. In Vitro.

[51] Cruz SL (2020). Minocycline prevents neuronal hyperexcitability and neuroinflammation in medial prefrontal cortex, as well as memory impairment caused by repeated toluene inhalation in adolescent rats. Toxicol. Appl. Pharmacol..

[52] Liu T (2017). NF-κB signaling in inflammation. Signal Transduct. Target. Ther..

[53] Mannan A (2021). Peroxisome proliferator-activated receptor-gamma (PPAR-ɣ): molecular effects and its importance as a novel therapeutic target for cerebral ischemic injury. Neurochem. Res..

[54] Jia J (2013). Regulation of iNOS expression by NF-κB in human lens epithelial cells treated with high levels of glucose. Invest. Ophthalmol. Vis. Sci..

[55] Shimizu S (2016). HMGB1-TLR4 Signaling Contributes to the Secretion of Interleukin 6 and Interleukin 8 by Nasal Epithelial Cells. Am. J. Rhinol. Allergy.

[56] Li Y (2015). Correlation of mechanical impact responses and biomarker levels: A new model for biomarker evaluation in TBI. J. Neurol. Sci..

[57] Rothaug, M., Becker-Pauly, C., & Rose-John, S. The role of interleukin-6 signaling in nervous tissue. Biochim. Biophys. Acta (BBA) Mol. Cell Res. **1863**(6), 1218–1227 (2016). 10.1016/j.bbamcr.2016.03.018 27016501

[58] Di Pietro V (2015). S100B and glial fibrillary acidic protein as indexes to monitor damage severity in an in vitro model of traumatic brain injury. Neurochem. Res..

[59] Kamel E-N, Shehata M (2008). Effect of toluene exposure on the antioxidant status and apoptotic pathway in organs of the rat. Br. J. Biomed. Sci..

[60] Aloe L (2015). Nerve growth factor: A focus on neuroscience and therapy. Curr. Neuropharmacol..

[61] Boucherie C (2008). In vitro evidence for impaired neuroprotective capacities of adult mesenchymal stem cells derived from a rat model of familial amyotrophic lateral sclerosis (hSOD1G93A). Exp. Neurol..

[62] Crisostomo PR (2008). Human mesenchymal stem cells stimulated by TNF-α, LPS, or hypoxia produce growth factors by an NFκB- but not JNK-dependent mechanism. Am. J. Physiol. Cell Physiol..

[63] Fuenzalida KM (2005). Peroxisome proliferator-activated receptor gamma is a novel target of the nerve growth factor signaling pathway in PC12 cells. J. Biol. Chem..

[64] Li X (2018). Intranasal administration of nerve growth factor promotes angiogenesis via activation of PI3K/Akt signaling following cerebral infarction in rats. Am. J. Transl. Res..

[65] Chen T (2019). MiR-27a promotes insulin resistance and mediates glucose metabolism by targeting PPAR-γ-mediated PI3K/AKT signaling. Aging (Albany NY).

[66] Di J (2017). Rap2B promotes angiogenesis via PI3K/AKT/VEGF signaling pathway in human renal cell carcinoma. Tumor Biol..

[67] Dick ALW (2015). Chronic intermittent toluene inhalation in adolescent rats results in metabolic dysfunction with altered glucose homeostasis..

[68] Crossin R (2019). The effect of adolescent inhalant abuse on energy balance and growth..

[69] Ercolano G (2021). PPARɣ drives IL-33-dependent ILC2 pro-tumoral functions. Nat. Commun..

[70] De Nuccio C (2011). Peroxisome proliferator-activated receptor γ agonists accelerate oligodendrocyte maturation and influence mitochondrial functions and oscillatory Ca2+ waves. J. Neuropathol. Exp. Neurol..

[71] Bloor AJC (2020). Production, safety and efficacy of iPSC-derived mesenchymal stromal cells in acute steroid-resistant graft versus host disease: a phase I, multicenter, open-label, dose-escalation study. Nat. Med..

[72] Ke X, Thakur A, Chen HJ (2022). Transdifferentiation meets next-generation biotechnologies. Stem J..

[73] Lian Q (2016). Directed differentiation of human-induced pluripotent stem cells to mesenchymal stem cells. Methods Mol. Biol..

[74] Stavely R, Nurgali K (2020). The emerging antioxidant paradigm of mesenchymal stem cell therapy. Stem Cells Transl. Med..

[75] Staff NP, Jones DT, Singer W (2019). Mesenchymal stromal cell therapies for neurodegenerative diseases. Mayo Clin. Proc..

