# Supplementary material for: Molecular evidence of the amelioration of toluene induced encephalopathy by human breast milk mesenchymal stem cells
Source: Sci Rep. 2022 Jun 2;12:9194. doi: 10.1038/s41598-022-13173-6 (PMC9163168; doi:10.1038/s41598-022-13173-6)
Supplement: Supplementary file 1 — Supplementary Tables. [file 41598_2022_13173_MOESM1_ESM.docx]

| ***Table: Forward and reverse of the primers for Peroxisome Proliferator-Activated Receptor-Gamma (PPAR-ɣ), nuclear factor kappa-B (NF-kB), and Interleukin-6 (IL-6) genes and the housekeeping glucose 6-phosphate dehydrogenase (G6PD) gene according to*** *(Khamis et al., 2021)****:*** | |
| --- | --- |
| ***Genes*** | ***Primer sequence*** |
| *PPAR-ɣ* | *Forward:5`- CCTGAAGCTCCAAGAATACC -3`*  *Reverse: 5`- GATGCTTTATCCCCACAGAC -3`* |
| *NF-kB* | *Forward:5`- CAGGACCAGGAACAGTTCGAA -3`*  *Reverse:5`- CCAGGTTCTGGAAGCTATGGAT -3`* |
| *IL-6* | *Forward:5`- ATATGTTCTCAGGGAGATCTTGGAA-3`*  *Reverse: 5`- GTGCATCATCGCTGTTCATACA -3`* |
| *G6PD* | *Forward:5`- GGCACAGTCAAGGCTGAGAATG -3`*  *Reverse:5`- ATGGTGGTGAAGACGCCAGTA -3`* |
